# Health Effects of Cut Gas Lines and Other Petroleum Product Release Incidents — Seven States, 2010–2012

**Published:** 2015-06-12

**Authors:** Ayana R. Anderson

**Affiliations:** 1 Division of Toxicology and Human Health Sciences, Agency for Toxic Substances and Disease Registry

Large mass casualty gas explosions and catastrophic oil spills are widely reported and receive considerable regulatory attention. Smaller, less catastrophic petroleum product releases are less likely to receive publicity, although study of these incidents might help focus and prioritize prevention efforts. To describe the causes and health impacts of petroleum product release incidents (including gas explosions and oil spills), the Agency for Toxic Substances and Disease Registry (ATSDR) analyzed 2010–2012 data from the National Toxic Substance Incidents Program (NTSIP). A total of 1,369 petroleum product release incidents were reported from seven states, resulting in 512 injuries and 36 deaths. Approximately one fourth of the incidents were associated with utilities, and approximately one fifth were associated with private vehicles or residences. Approximately 10% of petroleum product releases resulted from inadvertent damage to utility lines. Understanding the characteristics of acute petroleum product releases can aid the public and utility workers in the development of preventive strategies and reduce the morbidity and mortality associated with such releases.

Petroleum is refined to produce gasoline, heating oil, propane, and other fuels (1). If not managed properly, these products can adversely affect humans, wildlife, and the environment (2). Adverse health effects can include skin irritation, eye irritation, dizziness, headache, nausea and, and in extreme cases, death (2). Because petroleum is widely used, unintentional acute releases can occur almost anywhere.

In 2010, ATSDR established NTSIP to collect information useful for reducing morbidity and mortality associated with acute toxic substance releases.* State NTSIP partners collect information pertaining to acute petroleum and nonpetroleum releases and the public health effects of those releases and enter it into a web-based application. Acute nonpetroleum releases include but are not limited to any substance that, after release into the environment and upon exposure, ingestion, or inhalation, could cause morbidity or mortality. Nonpetroleum releases include chemical, biologic, radiologic and medical materials (3). However, NTSIP limits collection of information regarding releases of petroleum to those that result in an injury or a public health action (e.g., evacuation, shelter-in-place, alternative water usage, ban on fishing, health advisory, health investigation, prohibition against livestock or produce consumption, water intake shutdown or environmental sampling, and well survey). Additionally, NTSIP excludes petroleum-related incidents for which the only source of petroleum was the fuel tank of a vehicle involved in a crash.

During 2010–2012, seven states contributed data to NTSIP: Louisiana, New York, North Carolina, Oregon, Tennessee, Utah, and Wisconsin. To identify petroleum releases, ATSDR first searched the NTSIP system for “petroleum incidents” by searching on the chemical name variable for petroleum products listed in the 2010 NTSIP training manual (3). ATSDR then reviewed the comments and synopsis fields of the identified records to confirm that they described petroleum incidents. Descriptive statistical analyses comparing petroleum and nonpetroleum incidents were then performed.

NTSIP recorded 8,684 single-substance incidents during 2010–2012, of which 1,369 (15.8%) were petroleum-related. Of the 1,369 NTSIP petroleum-related incidents, 259 (18.9%) incidents included injuries (Table 1). In addition, 512 (15.1%) of the 3,399 persons injured in all NTSIP incidents were injured in petroleum incidents. The most commonly reported contributing factors for petroleum incidents were equipment failure (51.7%) and human error (40.2%). The remaining contributing factors were weather (4.3%), intentional or illegal acts (2.2%), and other factors (1.6%). Among the 1,369 petroleum incidents, 1,170 (85.5%) occurred in fixed facilities.

The utilities industry accounted for the greatest number of petroleum-release incidents (327 [23.9%]) (Table 2); most of these incidents (253 [77.4%]) were related to natural gas distribution. Of the utility releases, 131 (40.1%) involved lines damaged or cut because of errors by contractors, construction workers, or residents. A total of 14 (4.3%) of the 327 utility releases resulted in injuries, with a total of 27 persons injured (Table 2).

The second most commonly reported type of petroleum releases (296 [21.6%]) occurred in private vehicles and residences. These incidents were the most likely (105 [40.5%]) to result in injury and caused injuries to 236 persons (46.1%) (Table 2). Of the 105 petroleum-release incidents with injured persons, 59 (56.2%) incidents involved explosion or fire or both.

For both petroleum and nonpetroleum incidents, most injuries were to members of the general public, followed by employees (Table 3). Petroleum incidents resulted in a higher percentage of persons admitted to the hospital and deaths compared with nonpetroleum incidents (Table 3). The most commonly reported injuries for petroleum incidents were burns (32.5%) and trauma (24.6%). Petroleum incidents were less likely than nonpetroleum incidents to result in persons requiring decontamination (10.4% compared with 21.9%) (Table 3).

## Illustrative Case Reports

### Incident A

While a subcontractor was installing cable lines, he hit a 2-inch (51 mm) natural gas line. Natural gas leaked into the sewer system and into a townhouse, which exploded. One member of the public, two utility workers, and two firefighters were injured in the explosion. They had burns, trauma, and shortness of breath. All injured persons were treated at the hospital, but none were admitted. The gas was turned off, and hundreds of neighbors were evacuated for 24 hours.

### Incident B

A neighbor smelled natural gas and called utilities. Utility personnel investigated, but did not find a gas leak. Later, a nearby house exploded, injuring three members of the public. Two persons were admitted and treated at a hospital for burns. The third person died in the explosion. The area was evacuated for 6 days, affecting approximately 20 persons. The American Red Cross responded to provide assistance.

## Discussion

Petroleum release incidents have the potential to cause mass casualties and environmental contamination. In 2010, two incidents of acute, unintentional releases of petroleum products received prominent attention in the news media (4–7). One was a Pacific Gas and Electric gas line explosion in San Bruno, California. A 30-inch natural gas pipeline ruptured after reports from residents in the neighborhood stating they smelled gas. This release led to an explosion that left 35 homes burned, eight persons dead, and 30 more injured (6). The second incident was in Enbridge, Michigan, were a ruptured pipeline released more than 800,000 gallons of crude oil into the Kalamazoo River (5), resulting in serious environmental impacts and various adverse health effects (e.g., headache, nausea, and respiratory symptoms) in nearby residents (7).

NTSIP data from seven states for 2010–2012 indicate that petroleum incidents accounted for 15.8% of all toxic substance releases, and most of the petroleum incidents involved utilities. Nearly half of the utility incidents involved homeowners or construction contractors damaging or cutting lines. Petroleum releases caused by cut lines can be prevented if the public and construction professionals follow one simple precaution: call 811. Many underground utility pipes and conduits, but not all, are marked by signs above ground signaling their location. Each state has different rules and regulations governing digging, and some rules are more stringent than others. The telephone number 811 has been nationally designated to eliminate confusion over multiple “Call Before You Dig” numbers across the country. Dialing 811 connects callers with local centers that notify the appropriate local utilities, who then send crews to the requested site to mark the approximate location of underground lines at no charge (8).

Private vehicles and residences had the second greatest number of total petroleum release incidents, the greatest number of incidents involving injured persons, and the greatest total of injured persons. Many of these incidents were attributable to propane tank explosions, natural gas leaks, and gasoline misuse (e.g., using gasoline with charcoal grills and fireplaces).

The findings in this report are subject to at least three limitations. First, NTSIP data only include petroleum incidents that result in an injury or public health action; therefore, petroleum incident data are skewed toward higher percentages with injuries and evacuations. Second, because home incidents with no injury or public health action are not included in NTSIP, these data do not include all home incidents. Finally, with only seven states participating in NTSIP, no generalizations can be made regarding other states or data nationally.

What is already known on this topic?

Most petroleum products are highly flammable, and many can explode. Unintentional releases of petroleum products can cause significant morbidity, mortality, environmental damage, and financial loss.

What is added by this report?

During 2010–2012, a total of 1,369 unintentional petroleum product release incidents were reported by seven states to the National Toxic Substance Incidents Program. The incidents resulted in injuries to 512 persons and 36 deaths. Forty-six percent of the incidents were related to utilities, private residences, or private vehicles. The greatest number of petroleum release incidents resulted from cut utility lines or gas leaks, and burns were the most common type of injury.

What are the implications for public health practice?

The most common causes of petroleum release incidents are preventable. Contractors, construction workers, homeowners, and renters need to understand the potential health and environmental consequences of damaging gas lines when digging. Additionally, members of the public need to be made more aware of how to recognize gas leaks and of what can happen if they misuse petroleum products.

Because of the danger posed by petroleum incidents and their continuing occurrence, strategies to prevent releases are needed. Based on the NTSIP data, a comprehensive approach to construction worker training regarding ruptured line prevention might reduce petroleum release incidents and their health consequences. In addition, education is needed to inform the public regarding the safe use of petroleum products and the need to be able to recognize a gas leak and know what steps to take to prevent explosions and fires.

## Figures and Tables

**TABLE 1:** Number and percentage of reported petroleum release incidents compared with nonpetroleum and all reported single-substance incidents, by selected characteristics — National Toxic Substance Incidents Program (NTSIP) 2010–2012

Characteristic	Petroleum incidents		Nonpetroleum incidents		All reported NTSIP incidents	
		
	No.	(%*)	No.	(%*)	No.	(%*)
Incidents	1,369	—	7,315	—	8.684	—
Evacuations ordered	719	(52.5)	764	(10.5)	1,483	(17.2)
No. of evacuees	33,541	—	24,261	—	57,802	—
Incidents with injuries	259	(18.9)	1,114	(15.3)	1,373	(15.9)
No. of Injured persons	512	—	2,887	—	3,399	—
Shelter-in-place ordered	52	(3.8)	99	(1.3)	151	(1.7)

* Percentages calculated using the number of incidents in each column as denominators.

**TABLE 2:** Number and percentage of reported petroleum release incidents and injured persons, by type of industry or location — National Toxic Substance Incidents Program, 2010–2012

Industry or location	No. of incidents	(%)	No. of incidents with injuries	(%)	No. of injured persons	(%)
Utilities	327	(23.9)	14	(5.4)	27	(5.3)
Private residence, vehicle	296	(21.6)	105	(40.5)	236	(46.1)
Real estate	133	(9.7)	20	(7.7)	33	(6.4)
Educational services	86	(6.3)	4	(1.5)	4	(0.8)
Unknown	84	(6.1)	15	(5.8)	19	(3.7)
Transportation and warehousing	75	(5.5)	22	(8.5)	35	(6.8)
Retail trade	61	(4.5)	10	(3.9)	23	(4.5)
Manufacturing: paper, printing, chemicals, petroleum, leather, lumber, stone	52	(3.8)	8	(3.1)	23	(4.5)
Construction	36	(2.6)	6	(2.3)	8	(1.6)
Accommodation and food services	35	(2.6)	10	(3.9)	16	(3.1)
Health care and social assistance	33	(2.4)	5	(1.9)	12	(2.3)
Wholesale trade	31	(2.3)	11	(4.2)	15	(2.9)
Public administration	21	(1.5)	6	(2.3)	12	(2.3)
Arts, entertainment, and recreation	20	(1.5)	7	(2.7)	20	(3.9)
Administrative and support, waste management and remediation services	18	(1.3)	2	(0.8)	4	(0.8)
Mining	13	(0.9)	4	(1.5)	5	(1.0)
Other*	48	(3.5)	10	(3.9)	20	(3.9)
**Total**	**1,369**	**(100.0)**	**259**	**(99.9** † **)**	**512**	**(99.9** † **)**

* Includes Manufacturing: food, textile, metal, electric, transport, professional, and apparel; Professional: scientific and technical services; Agriculture: forestry, fishing, and hunting; Information; Finance and Insurance; Management of companies and enterprise; and Other services.

† Percentages do not equal 100 because of rounding.

**TABLE 3:** Number and percentage of persons injured from reported petroleum and nonpetroleum release incidents, by selected characteristics — National Toxic Substance Incidents Program, 2010–2012

Characteristic	Petroleum		Nonpetroleum	
	
	No.	(%)	No.	(%)
General public	277	(54.1)	1,290	(44.7)
Employee	159	(31.0)	1,089	(37.7)
Firefighter	63	(12.3)	168	(5.8)
Police officer	7	(1.4)	38	(1.3)
Unknown responder	2	(0.4)	13	(0.4)
Missing	4	(0.8)	8	(0.3)
Student	0	—	267	(9.3)
Hospital personnel	0	—	9	(0.3)
Employee response team	0	—	3	(0.1)
EMT personnel	0	—	2	(0.1)
**Total**	**512**	**(100.0)**	**2,887**	**(100.0)**
**Injured person disposition**				
Treated at hospital (not admitted))	217	(42.4)	1,760	(61.0)
Treated at hospital (admitted)	139	(27.1)	342	(11.8)
Treated on scene (First aid)	99	(19.3)	444	(15.4)
Died	36	(7.0)	109	(3.8)
On scene or on arrival at hospital	26	(72.2)	87	(79.8)
After arrival at hospital	10	(27.8)	22	(20.2)
Treated at hospital (admission unknown)	15	(2.9)	53	(1.8)
Observation at hospital, no treatment	2	(0.4)	53	(1.8)
Injury reported by official	1	(0.2)	69	(2.4)
See private physician in ≤24 hrs	0	—	26	(0.9)
Missing	3	(0.6)	31	(1.0)
**Injury type** *				
Burns	176	(32.5)	323	(8.4)
Thermal	137	(77.8)	84	(16.0)
Chemical	14	(8.0)	181	(56.0)
Both thermal and chemical	18	(10.2)	45	(13.9)
Unknown	7	(4.0)	13	(4.0)
Trauma	133	(24.6)	206	(5.3)
Nonchemical	98	(73.7)	121	(58.7)
Chemical	16	(12.0)	56	(27.2)
Both nonchemical and chemical	9	(6.8)	16	(7.8)
Unknown	10	(7.5)	13	(6.3)
Dizziness	82	(15.2)	873	(22.3)
Respiratory irritation	51	(9.4)	1,044	(26.7)
Headache	37	(6.8)	380	(9.7)
Other†	62	(11.5)	1,081	(27.7)
**Total**	**541**	**(100.0)**	**3,907**	**(100.0)**
**Persons decontaminated**				
Yes	53	(10.4)	630	(21.8)
No	451	(88.1)	2,202	(76.3)
Unknown	8	(1.6)	55	(1.9)

**Abbreviation:** EMT = emergency management technician.

* Some persons had multiple injuries.

† Other injuries included gastrointestinal, heat stress, eye irritation, heart problems, short of breath, and skin irritation.

## Footnotes

* Additional information available at http://www.atsdr.cdc.gov/ntsip.
